# Green synthesis of silver, iron and gold nanoparticles of lycopene extracted from tomato: their characterization and cytotoxicity against COLO320DM, HT29 and Hella cell

**DOI:** 10.1007/s10856-021-06489-8

**Published:** 2021-02-12

**Authors:** Kiran P. Shejawal, Dheeraj S. Randive, Somnath D. Bhinge, Mangesh A. Bhutkar, Sachin S. Todkar, Anjum S. Mulla, Namdeo R. Jadhav

**Affiliations:** 1 Department of Pharmaceutics Rajarambapu College of Pharmacy, Kasegaon, Walwa, Sangli, Maharashtra 415404 India; 2 Department of Pharmaceutical Chemistry Rajarambapu College of Pharmacy, Kasegaon, Walwa, Sangli, Maharashtra 415404 India; 3 Department of Pharmaceutics Bharati vidyapeeth College of Pharmacy, Kolhapur, Maharashtra 416013 India

## Abstract

Our study aimed at development of Silver, Iron and Gold nanoparticles of Lycopene isolated from tomato by using green synthesis technique and to evaluate its anticancer potential against colorectal and cervical cancer. Lycopene was extracted by benzene extraction method and the silver, iron and gold nanoparticles were developed by green synthesis method. 1% aqueous extract of isolated Lycopene was mixed with 1% solutions of AgNO_3_, FeCl_3_ and HAuCl_4_ solutions and incubated at ambient temperature for 3–4 h separately and observed for the color change which is an indicative of formation of the nanoparticles. The prepared nanoparticles were characterized by FTIR, SEM, XRD analysis and evaluated for their antimicrobial potential. The cytotoxicity studies were carried out by in vitro assay like MTT, SRB and Tryphan blue method against Colo 320 DM, HT 29, and Hella. SEM showed nanosized particles of 50–100 nm range, whereas no antimicrobial activity was exhibited by the prepared nanoparticles. In MTT assay the LyAgNP showed maximum 41.41 ± 0.4124% inhibition against COLO320DM, whereas LyGNP exhibited 41.47 ± 0.4469% inhibition against HT 29 and LyAgNP showed 40.9 ± 0.6908% inhibition against Hella cells. In SRB assay LyAgNP showed maximum 82.68 ± 1.1798% inhibition against COLO320DM, whereas LyGNP exhibited maximum 91.21 ± 0.2372% inhibition against HT29 and 87.98 ± 0.5878% inhibition against Hella cells. In tryphan blue assay against COLO320DM, HT29 and Hella cells, the maximum inhibition exhibited by the prepared nanoparticles were observed as LyGNP 83.45 ± 0.4694%, LyAgNP 88.05 ± 0.1870% and LyAgNP65.47 ± 0.4766%. We conclude that the developed nanoparticles of Lycopene exhibited potential anticancer activity against Colorectal and cervical cancer cell as compared with pure Lycopene.

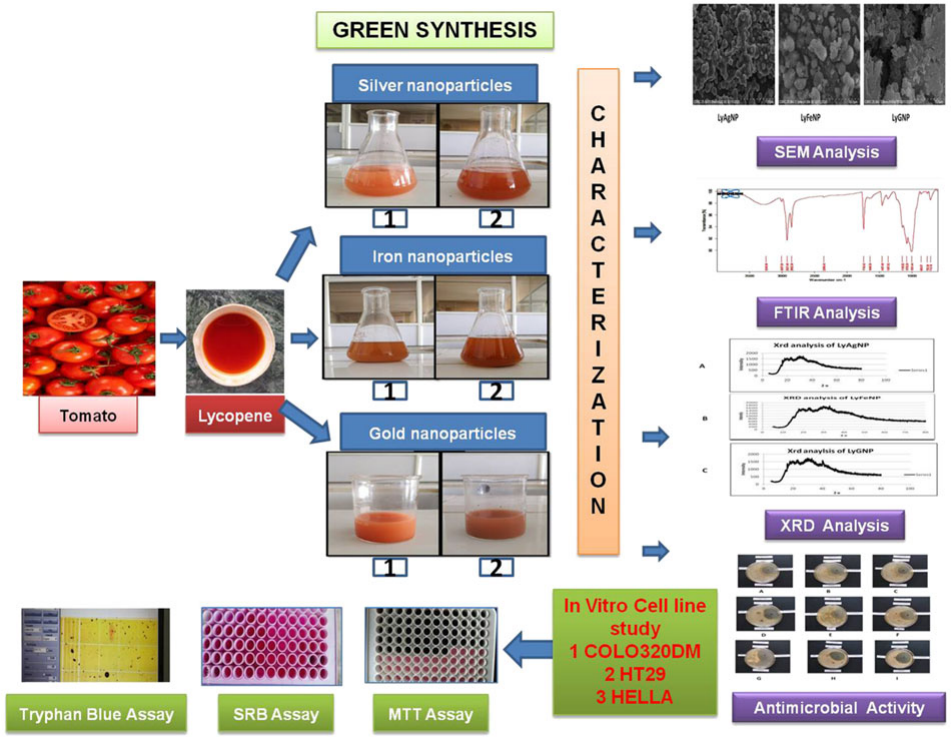

## Highlights

- The present research work is focused on green synthesis of Silver iron and gold nanoparticles of isolated lycopene from tomato.
- Our work aimed to assess scientifically the anticancer efficacy of the developed metal nanoparticles on in vitro colon and cervical cell lines.
- The results of the study revealed that developed nanoparticles exhibited significant cytotoxic activity against colon cancer and cervical cancer as compared with pure lycopene (phytoiconstituents).
- Use of the isolated phytoconstituents which are devoid of toxic effects of all other anticancer drugs and better absorption and cellular uptake due to its nano size.

## Introduction

Colorectal cancer is the 2nd most common reason of death from cancer [1]. The conventional chemotherapy is not much fruitful in its treatment as absorption of drug usually occurs in the stomach and intestine after its solubilization [2–4]. Nowadays, there is an increased interest in herbal drugs and remedies for the treatment of chronic diseases, including cancer [5]. A normally used approach is to begin pharmacological investigations with extraction of crude plant and subsequently isolate and characterize evaluate the phytoconstituents responsible for the pharmacological effect [6]. Scientists throughout the world have isolated numerous anticancer agents from plants [7]. However, the drug delivery system used for administering these phytoconstituent(s) to the patients remained obsolete leading to sub therapeutic efficiency in the treatment of disease [8]. Several studies have showed that greater intake of certain diets with fat or red meat and lower intake of diet rich in fruits and green vegetables is associated to a higher risk for colon cancer [9].

Carotenoids are important phytoconstituents that are considered as responsible for the protective effects of health by fruits and vegetables. Lycopene is also a carotenoid, a pigment mainly responsible for the characteristic red color of ripe tomato (*Lycopersicon esculentum*) and products of tomato, showed potential anticancer activity against several types of cancer cell lines [10, 11]. Its capability to act as a powerful antioxidant is thought to be responsible for protecting cells from oxidative damage and thereby diminishing the chances of chronic diseases [12]. The chances of a protective action of Lycopene on CRC is supported by the results obtained from a case-control study in Italy, which include a high tomato intake was constantly linked with a decreased risk of cancer of the gastrointestinal tract, including the colon along with rectum also [13]. Several findings have suggested that a high consumption of tomatoes and its products containing Lycopene may be protective against CVD and decrease hazard of several types of cancer, like prostate, breast, lung and digestive tract [14, 15]. Moreover, epidemiological documents have shown that group of people consuming traditional Mediterranean food with sufficient amounts of green vegetables, especially tomatoes, fruits, olive oil, grains, beans, and fish have reduced risk of chronic diseases like cancer. Also, Lycopene, a major constituent in tomato, showed potential anticancer activity against several types of cancer cell lines [10, 11].

Nanoparticles have been applicable as significant technology to deliver drugs, including peptides and proteins, vaccines and more newly nucleotides. Production of nanoparticles by using Green synthesis technique using plants has become an attractive area of research [16]. It offers advantages over the chemical and physical methods as they are cheaper, eco friendly and easily scrabbled up for huge scale synthesis and also there is no need of high energy, temperature, and hazardous chemicals. Metal nanoparticles (Au, Fe, and Ag) synthesis by using plant extract is much easier and advantageous over method of microbial cultures [17].

Thus, the intent of the present investigation was to develop Silver, Iron and Gold nanoparticles of Lycopene isolated from Tomato by using Green synthesis technique and to evaluate its anticancer potential against colorectal and cervical cancer.

## Materials and method

### Chemicals

Tomatoes required for extraction of Lycopene were purchased from a local market and after washing identified as *Lycopersicon esculentum* (*Solanaceae)*. Silver nitrate (AgNO_3_) and Ferric chloride (FeCl_3_) were obtained from Research Lab, Mumbai, and Maharashtra. All the other chemicals used in the study were of analytical grade. Chloroauric acid (HAuCl_4_) was purchased from Loba Chem, Kolhapur. Biomarker Lycopene was obtained as a gift sample from Influx healthcare Mumbai. Ciprofloxacin was obtained from Okasa Pharmaceuticals, Satara. Cell line COLO320DM, HT29 and Hella were procured from NCCS Pune Maharashtra.

## Methods

### Extraction and isolation of lycopene

#### Benzene extraction method

**Fig. 1:**
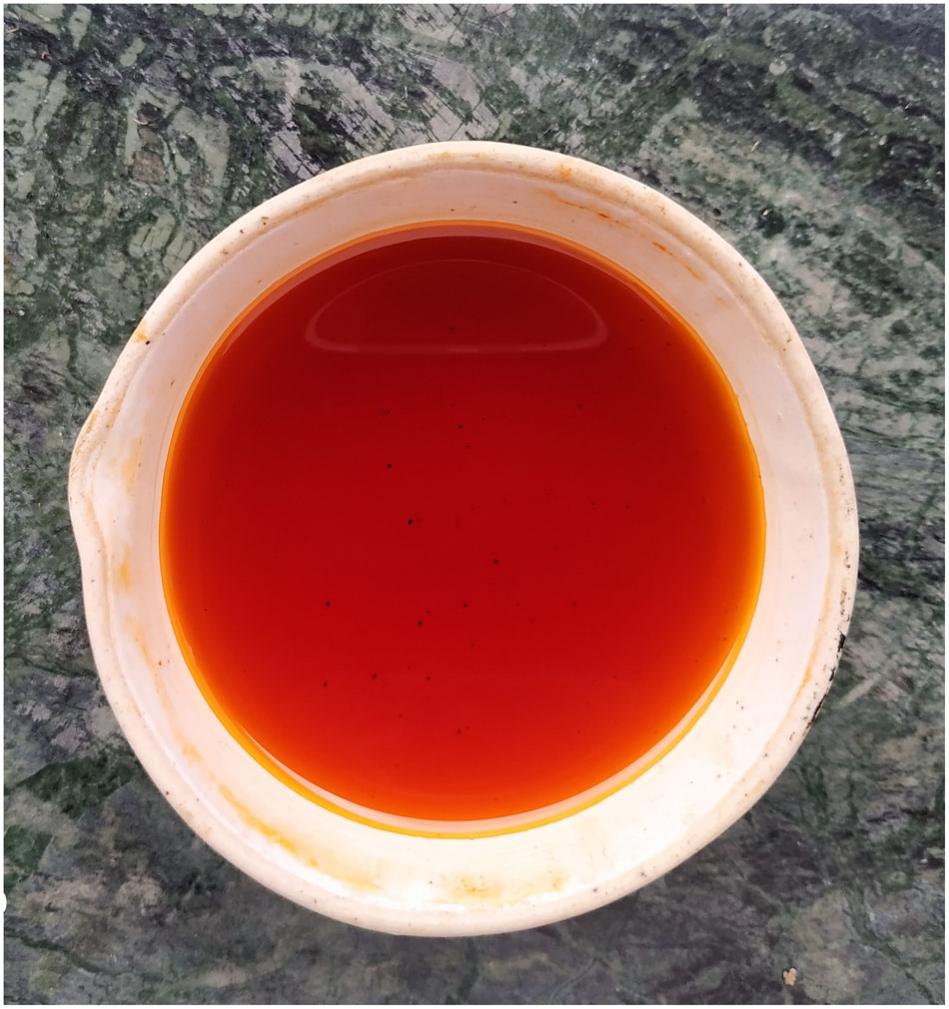
Isolated Lycopene from tomato

Paste of tomato was prepared by mixer crushing and 100 gm of it was taken in a 250 ml beaker. It was warmed and 30 ml of warm (40 °C) benzene was added about to it. Stirred and decanted the benzene layer. Again added 30 ml warm benzene, stirred and decanted the benzene. It was repeated for about five times. Then, Benzene was distilled off to obtain residue of Lycopene, as shown in (Fig. 1) recrystallized the residue by ether and weighed [18].

### Preparation of metallic nanoparticles

#### Synthesis of lycopene silver nanoparticles (LyAgNPs)

**Fig. 2:**
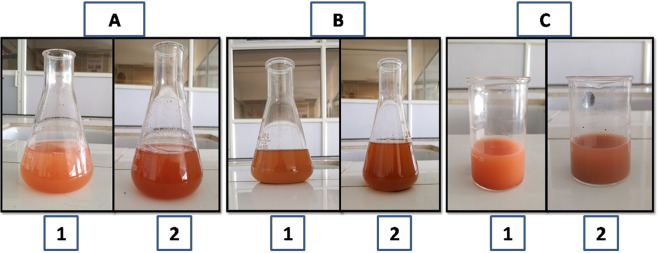
Synthesized nanoparticles (**A**) Silver (**B**) Iron (**C**) Gold

Lycopene extract was used for the synthesis of silver nanoparticles and silver nitrate (AgNO3) was used as a source of metal for synthesis of nanoparticles (LyAgNPs). Equal volumes of silver nitrate (1%) and Lycopene extract (1%) were vigorously mixed in the ratio of (1:1). The mixture was incubated for 3 h at room temperature. Change in color was observed by naked eye as shown in (Fig. 2A) and later analyzed by UV– Visible spectroscopy. The obtained nanoparticles (LyAgNPs) were purified through centrifugation at 10,000 *rpm* for 5 min, washed and dried in vacuum chamber for 24 h at 35 °C [19].

### Synthesis of lycopene iron nanoparticles (LyFeNPs)

LyFeNPs were synthesized using Lycopene extract (1%) extract and FeCl_3_ (1%) solution in accordance with the procedure mentioned by Phull et al. with minor modification. Equal volume (1:1) of 1% Lycopene extract and 1% ferric chloride (FeCl_3_) solutions were incubated at ambient temperature for 2–3 h to obtain LyFeNPs. Synthesis of LyFeNPs was detected by naked eye with a change of color from faint reddish to dark brown, as shown in (Fig. 2B) which was confirmed by UV spectroscopy [20].

### Synthesis of lycopene gold nanoparticles (LyAuNPs)

Chloroauric acid and the Lycopene extract were used for the synthesis of gold nanoparticles. 5 ml of aqueous extract of Lycopene was added to 10 mL of 1 mM aqueous Chloroauric acid (HAuCl_4_) solution in 250 mL Erlenmeyer flask and incubated in a rotary shaker at 150 rpm in dark. The change in color of colloidal solutions showed the formation of gold nanoparticles which is shown in (Fig. 2C) [21, 22].

### Characterization of nanoparticles

#### SEM analysis of LyAgNP, LyFeNP and LyGNP

Scanning Electron microscopy is an extra commonly used method of evaluation and morphological analysis at the nanometer to micrometer scale. Developed LyAgNP, LyFeNP, LyGNP were characterized using high resolution Scanning Electron Microscope (Schottky field emission scanning microscope SU5000). The samples were prepared by a simple drop coating of suspended gold solution on to an electric clean glass and allow the solvent to evaporate and the samples were kept to dry at room temperature. ImageJ software (Java2HTML Version 1.5) was used for the determination of the size of the developed nanoparticles.

#### FTIR spectroscopy analysis of LyAgNP, LyFeNP, LyGNP

To recognize the different biomolecules present in the extract of Lycopene and the phytocompounds capped on the Silver, Iron and Gold Nanoparticles after synthesis were analyzed by FTIR (Bruker Alpha Echo ATR). The spectrum was recorded in the range of 400–4000 cm^−1^

#### XRD analysis of LyAgNP, LyFeNP, LyGNP

The separated nanoparticles, were evaluated by XRD measurements using an XRD-6000 X-ray diffractometer Bruker D8 discover (voltage of 40 kV and 30 mA) with Cupper Kα radiation in θ–2θ configurations.

#### Antimicrobial activity of LyAgNP, LyFeNP and LyGNP

In vitro antimicrobial activity was carried out using the Agar Well Diffusion technique [23]. The sterile agar was inoculated with the bacteria culture (*S. aureus*, *P. aeruginosa*, and *E. coli*) for 48 h, at 37 °C. Antimicrobial activities were tested on nutrient medium against *S. aureus, P. aeruginosa* and *E. coli*, which are representative types of Gram positive and Gram negative organisms. Wells were bored by using a sterile borer, and standard solution (Ciprofloxacin) and test samples Lycopene, LyAgNP, LyFeNP and LyGNP (5 mg/mL was prepared by dissolving the test sample in DMSO) were placed into them (80 µL). Plates were kept for two hours in the refrigerator to enable prediffusion of the extracts into the agar. Next, the plates were incubated overnight (24–37 °C. The antimicrobial activity was determined by measuring the diameter of zone of inhibition recorded [24–28].

#### In vitro cytotoxicity studies of LyAgNP, LyFeNP and LyGNP by using MTT assay

Cell culture Human HT-29 cell, Colo 320 D and Hella celline was maintained in DMEM medium supplemented with 10% fetal bovine serum. The cells were plated at a density of 1 × 1 cells per well in a 96-well plate, and cultured for 24 h at 37 °C. The cells were subsequently exposed to 100 g/m. The plates were incubated for 24 h, and cell proliferation was measured by adding 10 µL of MTT (thiazolyl blue tetrazolium bromide) dye (5 mg/ml in phosphate-buffered saline) per well. The plates were incubated for a further 4 h at 37 °C in a humidified chamber containing 5% Co2 Formazan crystals formed due to reduction of dye by viable cells in each well were dissolved in 200 µl DMSO, and absorbance was read at 490 nm.

Finally, the percentage cytotoxicity of the compounds was calculated by using following formula.

$${\mathrm{Percent}}\;{\mathrm{Cytotoxicity}} = {\mathrm{Reading}}\;{\mathrm{of}}\;{\mathrm{control}} - {\mathrm{Reading}}\;{\mathrm{of}}\;{\mathrm{treated}}\;{\mathrm{cells/Reading}}\;{\mathrm{of}}\;{\mathrm{control}} \times {\mathrm{100}}$$

Since the absorbance was directly correlated with the number of viable cells, the percent viability was calculated from the absorbance. The IC50, the concentration of the drug at which 50% cell growth is inhibited, was calculated by the curve fitting of the cell viability data using Prism software.

#### In vitro cytotoxicity studies of LyAgNP, LyFeNP and LyGNP by using SRB assay

Human HT-29 cells, Colo 320 D and Hella cell line was maintained in DMEM medium supplemented with 10% fetal bovine serum. The cells were plated at a density of 1 × 10^4^ cells per well in a 96-well plate, and cultured for 24 h at 37 °C. The cells were subsequently exposed to 100 µg/ml compound. After drug incubation add 50 µL TCA (50%) and kept for 1 h in 4 °C then plate washed with TDW (triple distilled water) and air dry the plate. Then add 100 µLSRB dye in each well and kept for 30 min at room temperature. Again wash three times with 1% acetic acid and air dry the plate the add 200 µL tris buffer, and absorbance was read at 490 nm.

Finally, the percentage cytotoxicity of the compounds was calculated by using following formula.

$${\mathrm{Percentage}}\;{\mathrm{Cytotoxicity}} = {\mathrm{Reading}}\;{\mathrm{of}}\;{\mathrm{control}} - {\mathrm{Reading}}\;{\mathrm{of}}\;{\mathrm{treated}}\;{\mathrm{cells/Reading}}\;{\mathrm{of}}\;{\mathrm{control}} \times {\mathrm{100}}$$

#### In vitro cytotoxicity studies of LyAgNP, LyFeNP and LyGNP by using Tryphan blue assay

The dye exclusion test is used to determine the number of viable cells present in a cell suspension. It is based on the principle that live cells possess intact cell membranes that exclude certain dyes, such as Trypan blue, Eosin, or propidium, whereas dead cells do not. In this test, a cell suspension is simply mixed with dye and then visually examined to determine whether cells take up or exclude dye. In the protocol presented here, a viable cell will have a clear cytoplasm whereas a non viable cell will have a blue cytoplasm.

50 µL of cell lines of Human HT-29 cells, Colo 320 D and Hella were taken in microcentrifuge tube. Incubated them for 3 min then added 50 µL of all samples of nanoparticles in concentration of 100 µgmL^−1^ which were prepared by dissolving in Phosphate buffer pH 7.4 and DMSO incubated in CO_2_ incubator for 3 min and thereafter Tryphan blue (0.4%) 50 µL was added in each tube. Further incubated for 3 min in CO_2_ incubator and analyzed for total viable cells and non viable cells by using Nubars slide [29, 30].

### Statistical analysis

GraphPad Prism 8 for window 64 bit with version 8.0.1 (244) was used for the assessment of the statistical data of the antimicrobial and cytotoxicity activity. The results which were obtained obtained after performing the tests were analyzed by using One-way ANOVA with Dunnett’s post test analysis of variance. Mean ± SEM of all the calculated values were determined. A value of *P* < 0.05, 0.01, or 0.001 was considered statistically significant. ImageJ software (Java2HTML Version 1.5) has been used for the determination of the size of the prepared nanoparticles.

## Results and discussion

Nanoparticles have proved to be effective carriers for a variety of materials such as phytoconstituents, drug moieties, and functional dietary ingredients [31]. Conventional therapies used in cancer treatment suffer from several disadvantages and therefore there is an urgent need to explore the phytomedicine(s) which are effective and devoid of side effect(s). Moreover, effectiveness of the traditional medicine can be further modified if they are presented in nano form. Also, the targeting efficacy of the nanoparticles is markedly increased and drug release may be achieved at the desired site in an effective manner [31]. Conventional anticancer therapies in the treatment of colorectal cancer often exhibits discouraging results owing to the fact that the drug release occurs in the stomach and/or the intestinal region and inadequate amount of drug reaches to the colonic site. For the treatment of cervical cancer the drug bioavailability needs to be increased, which can be achieved by application of nanotechnology principles. Therefore, the said approach was taken into consideration during the plan of present study. Henceforth, the intent of the present investigation was to develop Silver, Iron, and Gold nanoparticles of Lycopene isolated from Tomato by using Green synthesis technique and to evaluate its anticancer potential against colorectal and cervical cancer. The study was aimed to assess the utility of Silver, Iron, and Gold nanoparticles of Lycopene isolated from Tomato in the treatment of cancer.

### SEM analysis of LyAgNP, LyFeNP and LyGNP

**Fig. 3:**
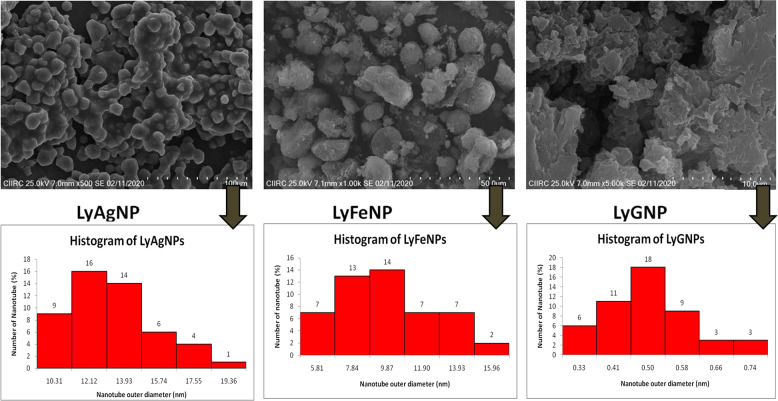
SEM images and Histogram of LyAgNP, LyFeNP, LyGNP

A scanning electron microscope was used to analyze the structure of the all three Ag, Fe, and Au nanoparticles that were formed and represented in Fig. 3. To find out the morphology, particle size and the periodicity of the synthesized nanoparticles by aqueous extract of Lycopene the SEM investigation was performed. The formed nanoparticles were found to be aggregated. This aggregation of the nanoparticles can be minimized or prohibited by increasing the concentration of the Lycopene extract. The average particle size was determined by ImageJ software and it was found to be in the range of 1–100 nm (Fig. 3).

### FTIR spectrum of LyAgNP, LyFeNP and LyGNP

**Fig. 4:**
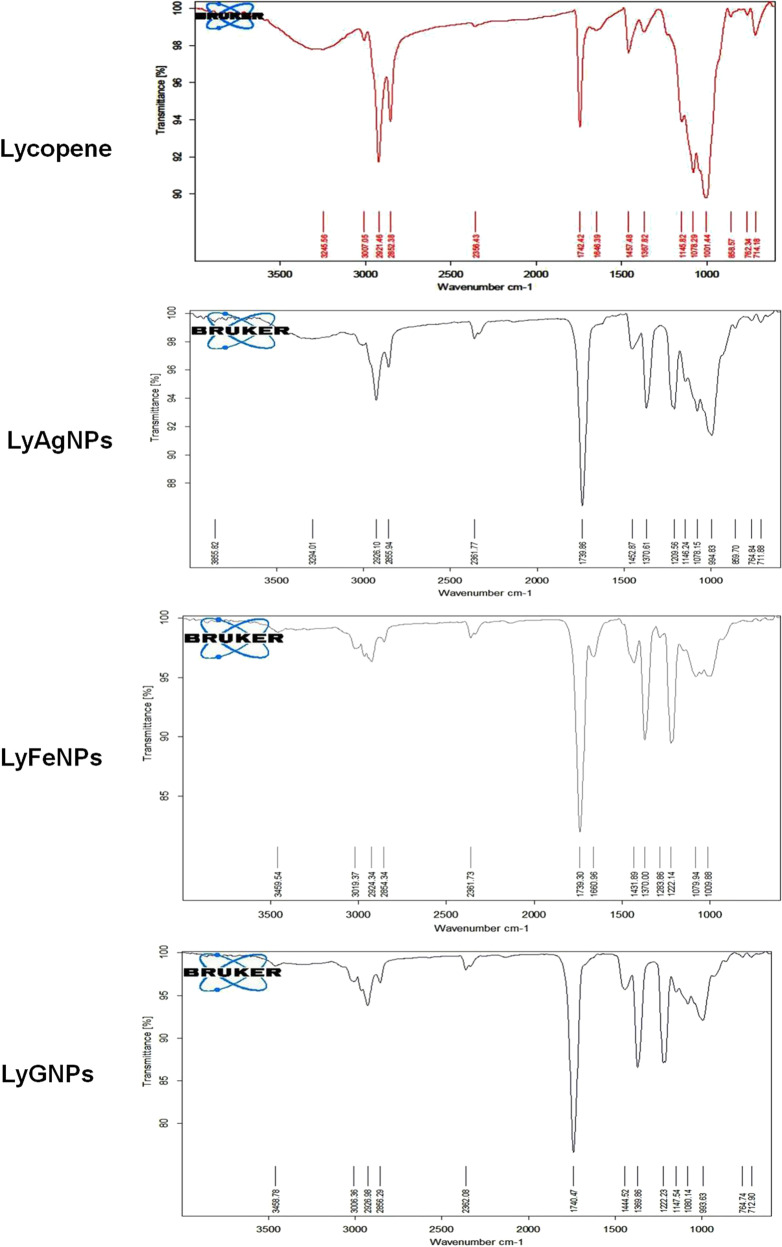
FTIR analysis of Lycopene, Silver nanoparticles LyAgNP, Iron LyFeNP, Gold LyGNP

In the FT-IR spectrum of Lycopene (Fig. 4), the C-Hstr(SP2), C-Hstr(SP3), C = Cstr, CH_2_(Bending), C-H(Trans OOP), R_2_C = CR bonds can be identified at 3007.05; 2921.46, 2852.38; 1646.49; 1457.48, 1367.82; 1145.82, 1078.29, 1001.44; and 714.18 cm^−1^ respectively, it play a role as the reductants in biosynthesis of GNPs [32], These characteristic vibrations after reduction of Au3+ ions were shifted to new peaks at 3006.36, 2926.98, 2856.29, 1740.47, 1444.52, 1369.96, 1147.54, 1080.14, 993.63 and 712.90 cm^−1^. Whereas the vibrations after reduction of Ag3+ ions were shifted to new peaks at 3006.45, 2926.10, 2855.94, 1739.96, 1452.87, 1370.61, 1146.24, 1078.15, 994.83 and 711.28 cm^−1^. Thus, 3019.37; 29224.34, 2854.34; 1739.30; 1431.89, 1370.00; 1079.94, 1009.88, and 710.24 cm^-1^ IR vibrations were observed after the reduction of Fe3 + ions. We noticed that C-Hstr(SP2) signals were enhanced when Lycopene was adsorbed on GNPs surface, and also CH2 deformation (1222.23 cm^−1^ in GNP; 1209.56 cm^−1^ in AgNPs; 1283.86 and 1222.14 cm^−1^ in FeNPs) peak instead of C-H scissoring. In addition, bio-reduction showed that the 1646.39, 1648.59 and 1660.96 cm^−1^ bands were suppressed in the GNP, AgNPs and FeNPs respectively. In addition, spectrum of the gold, silver and iron nanoparticle of Lycopene exhibited a strong peak at 1222.23, 1209.56 and 1283.86 cm^−1^ respectively, which were not observed in pure Lycopene, which indicates the formation gold, silver and iron nanoparticles of the Lycopene. Lycopene and NPs showed similar absorption bands, indicating that NPs might be stabilized by Lycopene. On the basis of the orange, yellow and blackish green color of the biomass and the groups suggested by FTIR analysis, it was hypothesized that Lycopene may be involved in gold, silver and iron nanoparticle synthesis [33–35].

### XRD spectrum of LyAgNP, LyFeNP and LyGNP

**Fig. 5:**
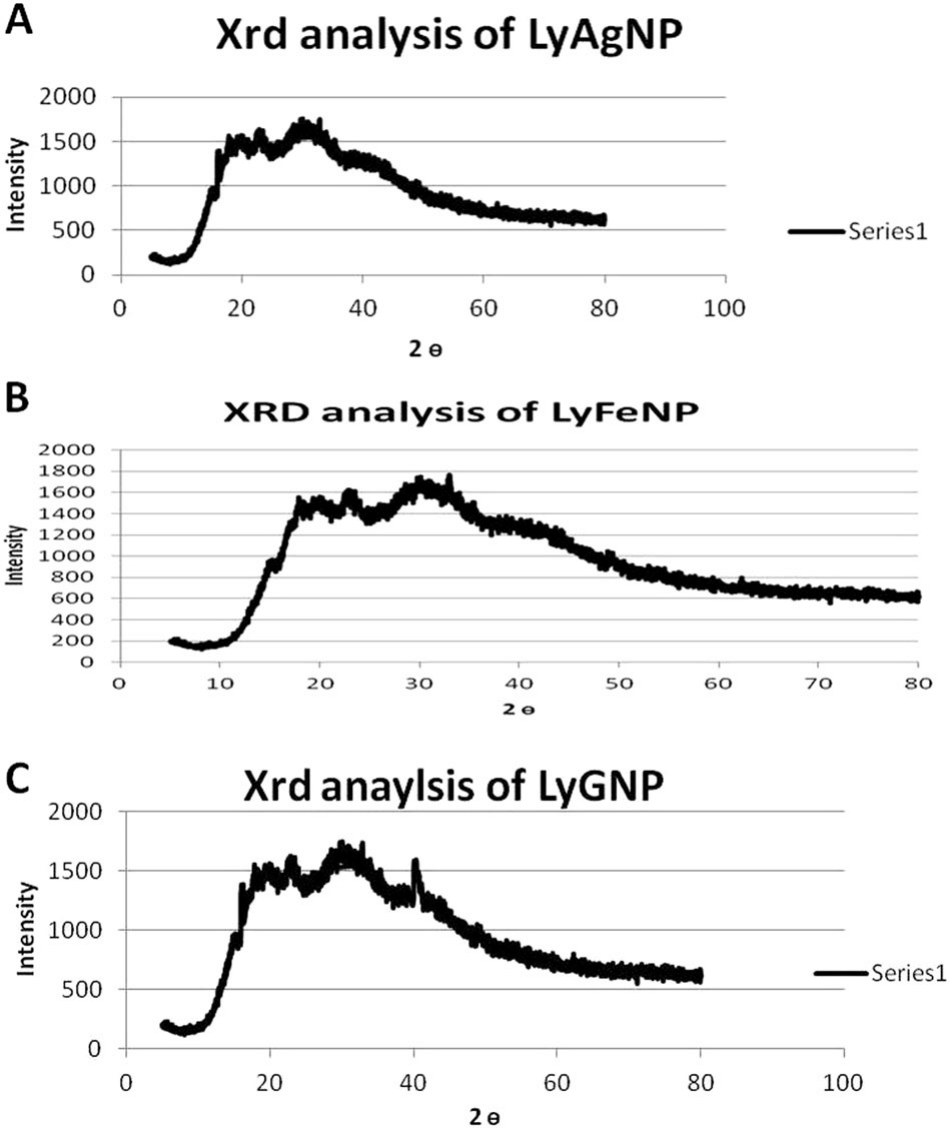
XRD analysis of Lycopene Silver nanoparticles LyAgNP, Iron LyFeNP, Gold LyGNP

XRD pattern of LyAgNP, LyFeNP, and LyGNP’ indicated that the particles are crystalline in nature (Fig. 5A–C), and strong diffraction peaks of LyAgNP’s were clearly observed at (110), (202), (219) and (310), and LyFeNPs (119) (251) (389) and 559) while LyGNP peaks were noted at (223), (412), (509) and (611). All the peaks obtained matched well with the Joint Committee on Powder Diffraction Standards (JCPDS) file no. 04–0783 of silver and 39–1346 of iron. The XRD pattern of the synthesized Silver, iron and gold nanoparticles from the aqueous extract of Lycopene is shown in Fig. 5. The diffraction peak at 2θ = 32° and subsequent higher order reflections can be indexed to the gold (Au) and other facets of gold nanoparticles. The XRD spectrum also reveal a weak peak around 2θ = 27°, which can be recognized to the phytoconstituent.

### Antibacterial activity of LyAgNP, LyFeNP and LyGNP

**Fig. 6:**
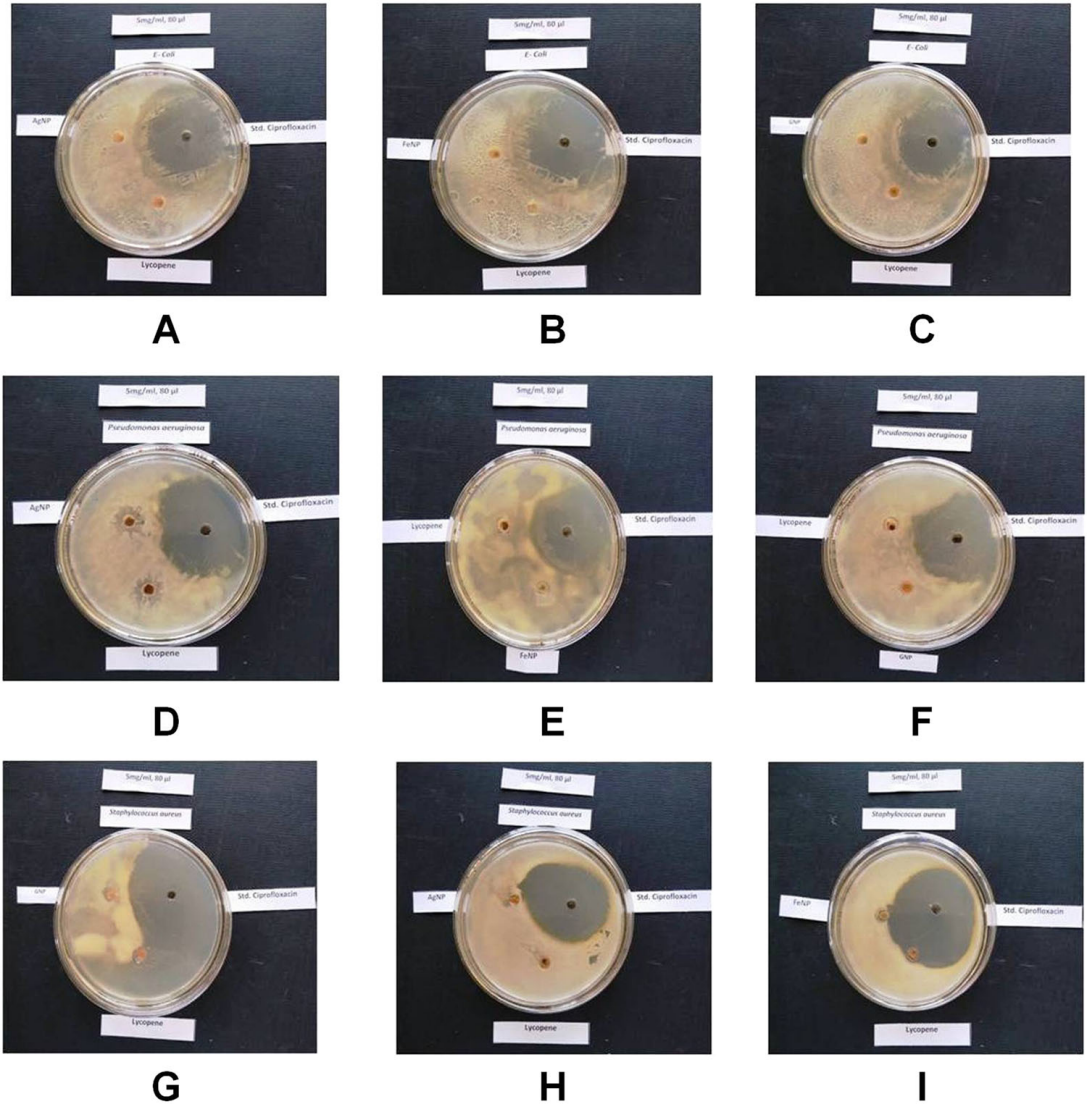
Antimicrobial Activity of Lycopene, Silver nanoparticles LyAgNP, Iron LyFeNP, Gold LyGNP

From the zone of inhibition it was observed that there is very little antimicrobial potential of pure Lycopene against *Pseudomonas aeruginosa* and *Staphylococcus aureus* with zones of inhibition 13 ± 0.11 mm and 06 ± 0.13 mm respectively whereas there is no antimicrobial potential shown by silver iron and gold nanoparticles against the selected microbial strains. The results are shown in Table 1 and Fig. 6.

**Table 1:** Antibacterial activity of LyAgNP, LyFeNP, LyGNP

Sr. no.	Sample name	Zone of Inhibition Diameter (mm) against the selected microorganisms		
		*Pseudomonas aeruginosa*	*Staphylococcus aureus*	*E. coli*
1	Lycopene Pure	13 ± 0.11	06 ± 0.13	0.00
2	Lycopene silver Nanoparticles	12 ± 0.38	0.00	0.00
3	Lycopene iron Nanoparticles	0.00	0.00	0.00
4	Lycopene gold Nanoparticles	0.00	0.00	0.00
5	Ciprofloxacin std.	45 ± 0.11	47 ± 0.13	42 ± 0.12

Values are expressed in triplicate mean SD

### Results of cytotoxicity of developed nanoparticles LyAgNP, LyFeNP, LyGNP

**Fig. 7:**
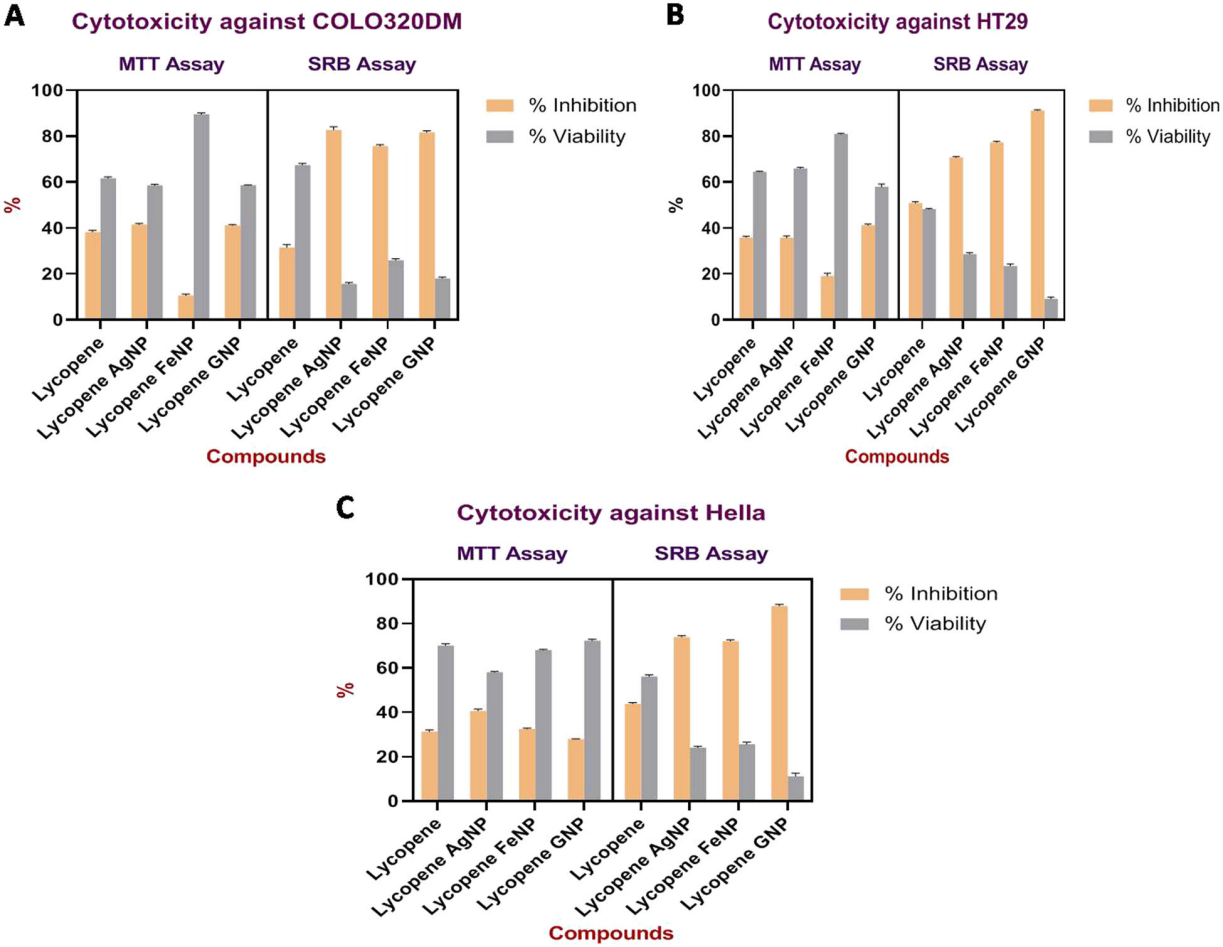
Cytotoxic Activity of Lycopene, Silver nanoparticles LyAgNP, Iron LyFeNP, Gold LyGNP (**A**) COLO320DM, (**B**) HT29, (**C**) Hella cell lines

The results of cytotoxicity assay of LyAgNP, LyFeNP, LyGNP by using MTT Assay against COLO 320 DM, HT29 and Hella cells are shown in Table 2 and Fig. 7. LyAgNP showed 41.41 ± 0.4124% inhibition against COLO320DM as compared to other nanoparticles. Whereas LyGNP showed 41.47 ± 0.4469% inhibition against HT 29 and LyAgNP exhibited 40.9 ± 0.6908% inhibition against Hella cells.

**Table 2:** Results of cytotoxicity of LyAgNP, LyFeNP, LyGNP by using MTT Assay against COLO 320 DM, HT29 and Hella

Sr no	Cell line	Compound	Mean	% inhibition	% viability
1	COLO 320 DM	Control	0.384		
2		Lycopene	0.237	38.22 ± 0.5688	61.59 ± 0.5589
3		Lycopene AgNP	0.225	41.41 ± 0.4124	58.56 ± 0.3642
4		Lycopene FeNP	0.343	10.55 ± 0.5008	89.54 ± 0.4337
5		Lycopene GNP	0.225	41.13 ± 0.2706	58.58 ± 0.0946
1	HT 29	Control	0.394		
2		Lycopene	0.254	36.03 ± 0.4874	64.31 ± 0.2168
3		Lycopene AgNP	0.204	35.43 ± 0.6740	65.94 ± 0.3569
4		Lycopene FeNP	0.319	19.23 ± 0.9458	81.1 ± 0.2510
5		Lycopene GNP	0.232	41.47 ± 0.4469	57.83 ± 0.9295
1	Hella	Control	0.325		
2		Lycopene	0.226	31.26 ± 0.6340	70.28 ± 0.7064
3		Lycopene AgNP	0.189	40.9 ± 0.6908	58.25 ± 0.2719
4		Lycopene FeNP	0.220	32.65 ± 0.3332	67.96 ± 0.1932
5		Lycopene GNP	0.235	19.5 ± 0.1465	72.03 ± 0.5289

Values are expressed in triplicate mean ± SD

In case of SRB assay the results obtained are quite different as stated in Table 3 and Fig. 7. In this assay LyAgNP showed maximum 82.68 ± 1.1798% inhibition against COLO320DM, whereas LyGNP showed maximum 91.21 ± 0.2372 % inhibition against HT29 and 87.98 ± 0.5878% inhibition against Hella cells.

**Table 3:** Results of cytotoxicity of LyAgNP, LyFeNP, LyGNP by using SRB Assay against COLO 320DM, HT 29 and Hella

Sr no	Cell line	Compound	Mean	% inhibition	% viability
1	COLO 320 DM	Control	0.252		
2		Lycopene	0.172	31.53 ± 0.9385	67.45 ± 0.5718
3		Lycopene AgNP	0.040	82.68 ± 1.1798	15.62 ± 0.4579
4		Lycopene FeNP	0.063	75.69 ± 0.4946	25.86 ± 0.6100
5		Lycopene GNP	0.044	81.61 ± 0.6518	18.04 ± 0.4467
1	HT 29	Control	0.247		
2		Lycopene	0.120	50.84 ± 0.4828	48.27 ± 0.2384
3		Lycopene AgNP	0.078	70.62 ± 0.3823	28.49 ± 0.4415
4		Lycopene FeNP	0.058	77.09 ± 0.4269	23.99 ± 0.7189
5		Lycopene GNP	0.021	91.21 ± 0.2372	9.19 ± 0.6121
1	Hella	Control	0.301		
2		Lycopene	0.167	43.96 ± 0.4158	56.16 ± 0.5328
3		Lycopene AgNP	0.076	74.1 ± 0.4766	24.5 ± 0.5307
4		Lycopene FeNP	0.081	72.39 ± 0.4845	25.88 ± 0.7597
5		Lycopene GNP	0.034	87.98 ± 0.5878	11.5 ± 1.1368

Values are expressed in triplicate mean ± SD

Tryphan blue assay against COLO320DM, HT29 and Hella cells showed that LyGNP exhibited 83.45 ± 0.4694%, LyAgNP showed 88.05 ± 0.1870% and LyAgNP revealed 65.47 ± 0.4766% inhibition as shown in Table 4.

**Table 4:** Results of cytotoxicity of LyAgNP, LyFeNP, LyGNP by using tryphan blue assay

Sr. no	Drug	Colo 320DM		HT29		Hella	
		% inhibition	% viability	% inhibition	% viability	% inhibition	% viability
1	Lycopene	62.99 ± 0.3253	37.82 ± 0.2619	57.94 ± 0.6343	41.14 ± 0.7760	61.25 ± 0.2658	38.75 ± 0.3899
2	Lycopene AgNP	79.44 ± 0.4967	21.28 ± 0.2902	88.05 ± 0.1870	12.08 ± 0.0946	65.47 ± 0.4766	36.05 ± 0.7071
3	Lycopene FeNP	64.8 ± 0.4172	33.93 ± 0.6697	67.86 ± 0.2661	30.76 ± 0.7734	64.76 ± 0.4115	35.5 ± 0.4215
4	Lycopene GNP	83.45 ± 0.4694	16.31 ± 0.3922	80.72 ± 0.8134	18.74 ± 0.4422	57.51 ± 0.4805	42.4 ± 0.6191

Values are expressed in triplicate mean ± SD

## Conclusion

The biosynthesis of silver, iron, and gold nanoparticles using the extracted lycopene from tomato was economical, non-toxic, and eco-friendly. The synthesized nanoparticles were stable due to the reducing and capping nature of phyto-constituents. Nanoparticles showed good cytotoxic activity against colon cancer cells COLO320DM, HT29 and cervical cancer Hella cell line also may provide as a potential anticancer treatment for cancer therapy. Based on the results of in vitro assay methods namely MTT, SRB and tryphan blue, we conclude that the developed nanoparticles were significant than the lycopene owing to their nano size. With this research we have provided an important base for the use of plant based or phytoconstituent(s) in the form of nanoparticles for the treatment of human colon and cervical cancer.

## References

[1] Vitiani LR, Lombardi DG, Pilozzi E, Biffoni M, Todaro M, Peschle C, et al. Identification and expansion of human colon-cancer-initiating cells. Nature. 2007;445:111–5. 10.1038/nature05384 17122771

[2] Toshihiko K, Ronald L, Vernon ES, Gary JK, Robert BK, Chinthalapally VR, et al. Chemopreventive Effect of Curcumin, a Naturally Occurring Anti-Inflammatory Agent, during the Promotion/Progression Stages of Colon Cancer. Cancer Res. 1999;59:597–601. 9973206

[3] Paradkar M, Amin J (2018). Formulation development and evaluation of colon targeted delayed release methotrexate pellets for the treatment of colonic carcinoma. Braz J Pharm Sci..

[4] Alberto F, Valeria Z, Antonio M, Francesca C, Anna VM (2017). Mediterranean Diet and Colorectal Cancer: a systematic review. Nutrition.

[5] Randive DS, Bhinge SD, Bhutkar MA, Shejwal KP, Patil PD, Mane SA (2019). Hypoglycemic effects of Lagenaria siceraria, Cynodon dactylon and Stevia rebaudiana extracts”. J Herbmed Pharmacol..

[6] Randive DS, Sayyad SF, Bhinge SD, Bhutkar MA (2016). Preparation of Arjunāriṣṭa Using Microbes Isolated from Woodfordia fruticosa Flowers (Dhayati). Anc Sci Life.

[7] Uma YR, Chang-S K, Lee JI, Kim YA, Nam TJ, Seo Y (2010). Evaluation of chemical constituents from Glehnia littoralis for antiproliferative activity against HT-29 human colon cancer cells. Process Biochem.

[8] Kattamanchi G, Kalakonda SN, Rao BG (2017). Phospholipid Complex Technique for Superior Bioavailability of Phytoconstituents. Adv Pharm Bull.

[9] Charepalli V, Reddivari L, Vadde R, Walia S, Radhakrishnan S, Jairam KPV (2016). Eugenia jambolana (Java Plum) Fruit Extract Exhibits Anti-Cancer Activity against Early Stage Human HCT-116 Colon Cancer Cells and Colon Cancer Stem Cells. Cancers.

[10] Tang FY, Pai H, Wang XD (2011). Consumption of Lycopene Inhibits the Growth and Progression of Colon Cancer in a Mouse Xenograft Model. J Agric Food Chem..

[11] Lin MC, Wang FY, Kuo YH, Tang FY (2011). Cancer Chemopreventive Effects of Lycopene: Suppression of MMP-7 Expression and Cell Invasion in Human Colon Cancer Cells. J Agric Food Chem..

[12] Rao AV, Waseem Z, Agarwal S (1998). Lycopene content of tomatoes and tomato products and their contribution to dietary lycopene. Food Res Int.

[13] Erhardt JG, Meisner C, Bode JC, Bode C (2003). Lycopene,-carotene, and colorectal adenomas, Printed in USA. © 2003 American Society for Clinical Nutrition. Am J Clin Nutr..

[14] Adetayo OO, Rotimi EA (2005). The anticarcinogenic and anti-atherogenic effects of lycopene: a review. Trends Food Sci Technol.

[15] Cha JH, Kim WK, Ha AW, Kim MH, Chang MJ (2017). Anti-inflammatory effect of lycopene in SW480 human colorectal cancer cells. Nutr Res Pract.

[16] Patil OA, Patil IS, Mane RU, Randive DS, Bhutkar MA, Bhinge SD (2018). Formulation optimization and evaluation of Cefdinir nanosuspension using 23 Factorial design. Marmara Pharm J..

[17] Prabhu S, Poulose EK (2012). Silver nanoparticles: mechanism of antimicrobial action, synthesis, medical applications, and toxicity effects. Int Nano Lett.

[18] Lilwani S, Nair V (2015). Extraction and Isolation of Lycopene Form Various Natural Sources. J Biotechnol Biochem.

[19] Zargar M, Hamid AA, Bakar FA, Shamsudin MN, Shameli K, Jahanshiri F, et al. Green Synthesis and Antibacterial Effect of Silver Nanoparticles Using Vitex Negundo L. Molecules. 2011;16:6667–76. 10.3390/molecules16086667 PMC6264443 25134770

[20] Raju AI, Bharadwaj MS, Prem K, Satyanandam K (2016). Green Synthesis of Iron Nanoparticles using Albizia lebbeck leaves for Synthetic Dyes Decolorization, International Journal of Science. Eng Technol Res.

[21] Elavazhagan T, Arunachalam KD (2011). Memecylon edule leaf extractmediated green synthesis of silver and gold nanoparticles. Int J Nanomed.

[22] Arunachalam KD, Arun LB, Annamalai SK, Arunachalam AM (2014). Biofunctionalized Gold Nanoparticles Synthesis from Gymnema Sylvestre And its preliminary anticancer activity. Int J Pharm Pharm Sci..

[23] Bhinge SD, Hogade MG, Savali AS, Hariprassana RC, Magdum CS (2013). Antibacterial activity of bark extract of Ficus glomerataroxb against some Gram positive and Gram negative bacteria. Indian Drugs.

[24] Bhinge SD, Randive DS, Bhutkar MA, Wadkar GH, Kamble SY, Kalel PD, et al. Formulation and evaluation of polyherbal gel containing extracts of Azadirachta indica, Adhatoda vasica, Piper betle, Ocimum tenuiflorum and Pongamia pinnata. J Res Pharm. 2019;23:44–54. 10.12991/jrp.2018.107

[25] Bhinge SD, Randive DS, Bhutkar MA, Wadkar GH, Todkar SS, Kakade PM, et al. Formulation Dev evaluation antimicrobial polyherbal gel Annales Pharmaceutiques Françaises. 2017;75:349–58. 10.1016/j.pharma.2017.04.006 28583316

[26] Randive DS, Bhinge SD, Bhutkar MA, Joshi SR, Patil PD, Shejawal KP, et al. Formulation and evaluation of Herbal cough remedy from extract of Calendula officinalis L. Indian Drug. 2020;57:52–58.

[27] Randive DS, Bhinge SD, Jadhav NR, Bhutkar MA, Shirsat MK (2020). Assessment of antimicrobial efficacy of kohl/kajal prepared by different indian methods against selected microbial strains. Int J Curr Pharm Res.

[28] Randive DS, Bhinge SD, Jadhav NR, Bhutkar MA, Shirsat MK. Carbon Based Kajal Formulations: Antimicrobial Activity and Feasibility as a Semisolid Base for Ophthalmics. J Pharm Res Int. 2020;32:62–74.

[29] Mosmann T (1983). Rapid colorimetric assay for cellular growth and survival: application to proliferation and cytotoxicity assays. J Immunol Methods.

[30] Shejawal KP, Randive DS, Bhinge SD, Bhutkar MA, Wadkar GH, Jadhav NR (2020). Green synthesis of Silver and iron nanoparticles of isolated proanthrocynidine: its Characterization, antioxidant, antimicrobial and cytotoxic activities against COLO320DM and HT29. J Genet Eng Biotechnol.

[31] Chavane R, Bhinge SD, Bhutkar MA, Randive DS, Wadkar GH, Todkar SS, et al. Characterization, antioxidant, antimicrobial and cytotoxic activities of green synthesized silver and iron nanoparticles using alcoholic Blumea eriantha DC plant extract, Material Today. Communications. 2020;24:101320. 10.1016/j.mtcomm.2020.101320

[32] Aghel N, Ramezani Z, Amirfakhrian S (2011). isolation and quantification of lycopene from tomato cultivated in dezfoul, iran, Jundishapur. J Nat Pharm Products.

[33] Sowani H, Mohite P, Damale S, Kulkarni M, Zinjarde S (2016). Carotenoid stabilized gold and silver nanoparticles derived from the Actinomycete Gordonia amicalis HS-11 as effective free radical scavengers. Enzym Microb Technol.

[34] Nguyen HYT, Vo BHT, Nguyen LTH (2013). “Extracts of Crinum latifolium inhibit the cell viability of mouse lymph oma cell line EL4 and induce activation of anti-tumour activity of macrophages in vitro. J Ethnopharmacol.

[35] Vo TT, Nguyen TTN, Huynh TTT, Vo TTT, Nguyen TTN, Nguyen DT, et al. Biosynthesis of Silver and Gold Nanoparticles Using Aqueous Extract from Crinum latifolium Leaf and Their Applications Forward Antibacterial Effect and Wastewater Treatment. J Nanomater. 2019;14, 10.1155/2019/8385935

